# Characterization of volatile aroma compounds in litchi (Heiye) wine and distilled spirit

**DOI:** 10.1002/fsn3.2361

**Published:** 2021-09-18

**Authors:** Lili Zhao, Shili Ruan, Xiangke Yang, Qiling Chen, Kan Shi, Ke Lu, Ling He, Shuwen Liu, Yangbo Song

**Affiliations:** ^1^ College of Enology Northwest A&F University Yangling China; ^2^ Technical Center of Yantai Changyu Group Co., Ltd Yantai China; ^3^ Agriculture and Animal Husbandry College Qinghai University Xining China; ^4^ College of Horticulture Northwest A&F University Yangling China

**Keywords:** litchi distilled spirit, litchi wine, OAVs, SBSE‐GC/MS, volatile compounds

## Abstract

This study used litchi (Heiye) wine and distilled spirit as raw experimental materials to analyze the volatile aroma compounds. Qualitative and quantitative determination of aromatic components was studied using stir bar sportive extraction (SBSE) and gas chromatography coupled to mass spectrometry (GC/MS). Results indicated that a total of 128 different types of aroma compounds were observed, which belonged to six chemical groups, including 39 esters, 16 alcohols, 16 acids, 22 terpenes, 17 aldehydes and ketones, and 18 other compounds. In particular, esters were the highest among all six categories and represented approximately 52% of the total flavor component content in litchi distilled spirit. The odor activity values (OAVs) revealed 22 types of aroma compounds with OAVs >1 in this test. It is possible that the produced litchi distilled spirit had a stronger varietal character due to the increased concentrations and OAVs of β‐damascenone, linalool, ethyl butyrate, ethyl isovalerate, ethyl caproate, trans‐rose oxide, and cis‐rose oxide. Taking the OAVs into account, we evaluated the characteristic aromas for litchi wine and litchi distilled spirit.

## Graphical abstract

It is possible that the produced litchi distilled spirit had a stronger varietal character due to the increased concentrations and OAVs of β‐damascenone, linalool, ethyl isovalerate, ethyl butyrate, ethyl caproate, trans‐rose oxide, and cis‐rose oxide since these compounds are known to play a decisive fruit and flower aroma in the distilled spirit.

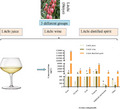

**Highlights**

- The naturally occurring litchi (Heiye) has potential applications in wine products, particularly in distilled spirit.
- 128 different types of aroma compounds were identified in this study.
- Alcoholic fermentation and distilled spirit process lead to a series of by‐products.
- It is possible that the produced litchi distilled spirit had a stronger varietal character.

## 1 INTRODUCTION

Litchi (*Litchi chinensis* Sonn.) is an important fruit crop originated in China and has earned its popularity worldwide due mainly to its charming aroma, unique taste, and possible health benefits. In recent years, litchi cultivation has been distributed primarily for countries in Southeast Asia, such as China, India, Thailand, and Vietnam which are the leading litchi‐producing countries in the world, among which China is the largest producing country (Pareek, 2016). Several cultivars in the west of Guangdong region have a long history of cultivation and account for approximately one‐third of all fruit exports in China, while others are relatively new (Emanuele et al., 2017; Xiong et al., 2018). Litchi is suitable for producing fruit wine, with its high sugar content (up to 19.2%) and rose‐floral and citrus‐like aroma (Chen et al., 2014). Various physiological functions have also been reported for litchi, such as cancer preventive, antioxidant activity, antimicrobial, anti‐inflammatory activities, and so on (Emanuele et al., 2017; Kilari & Putta, 2016; Varzakas et al., 2016). These days, the fermented fruit alcohol industry shows increasing interest in functional food, which displays an additional function related to disease prevention or health promotion. The naturally occurring litchi is a prime candidate. A wide range of litchi cultivars and growing climates in addition to orchard management practices allow for countless variables in starting fruit material. Likewise, prefermentation treatments of fruit and juice, fermentation management, and postfermentation treatments of litchi wine will also have impacts on final product quality. Li et al. (2012) found a 3.2‐fold difference in phenolic content between the highest and lowest litchi varieties, Heiye and Chanchutou, respectively. However, litchi pericarp browning and pulp decay lead to its short shelf life; it is liable to lose its attractive feature and rapidly goes unpleasant once harvested from trees (Zhao et al., 2020). Therefore, much attention has been paid to postharvest quality preservation via various approaches and further processing, including the production of litchi wine and distilled spirit, aiming to keep its excellent characteristics for a more extended period.

Today, the consumption of distilled spirit has become increasingly and wildly popular in the world. Globally, famous distilled spirits such as whiskey, brandy, and rum occupy most of the domestic alcohol market because of recognition as high‐quality products (Lee et al., 2018). And there have been numerous studies regarding the volatile aroma compounds in fermented wine and distilled spirit. Sánchez‐Palomo et al. (2019) identified 77 volatile compounds in Chelva wines and the Chelva grapes variety cultivated in La Mancha region presents an excellent aroma potential and a complex sensory profile. Chen et al. (2019) found more aroma‐active areas with flavor dilution (FD) factors of ≥64 in the aged rice wine than in the young rice wine, and the odor activity values (OAVs) revealed 33 aroma compounds with OAVs of ≥1 in young or aged rice wine. Lee et al. (2018) analyzed the flavor of aged spirits made from sweet potato and rice by gas chromatography‐mass spectrometry. Zheng et al. (2014) studied the fundamental volatile aroma compound changes in sweet Hongqu glutinous rice wine (a select type of rice wine) by headspace solid‐phase microextraction (HS‐SPME) and gas chromatography‐olfactometry (GC‐O). Wang et al. (2018) detected 71 kinds of aroma components in five distilled spirit (made from main grape varieties). Among them, the contents of esters, alcohols, and acids were higher. Yi et al. (2010) analyzed volatile compounds in liquor distilled from mash produced using Koji or Nuruk under reduced or atmospheric pressure. Tang et al. (2019) observed a total of 97 volatiles (38 in common) in lychee wine during fermentation, including 39 terpenoids, 5 alcohols, 22 esters, 4 acids, 7 alkenes, 4 ketones and ethers, 3 sulfur compounds, and others. Chen and Liu (2016) found 84 volatiles and 88 volatiles in the AF (alcoholic fermentation) and MLF (malolactic fermentation) litchi wines, respectively. However, there is little research regarding the flavor of litchi distilled spirit, particularly those made from litchi (Heiye) as one of the raw materials.

The purpose of this study was to explain the difference in aroma compounds between litchi (cv. Heiye) wine and distilled spirit by SBSE‐GC/MS technology and to objectively evaluate the contribution of aroma compounds to litchi wine and distilled spirit through the calculation of OAVs. Based on these results, the study may improve our understanding of the essence of the aroma difference between litchi wine and litchi distilled spirit. It can provide a substantial basis for further research into the control of flavor during the distilled process for litchi distilled spirit.

## 2 MATERIALS AND METHODS

### 2.1 Litchi materials

Two varieties of litchi (cvs. Heiye an Guiwei) were from Maoming, Guangdong Province, China, 2013 vintage, at their optimum point of maturity and sound quality without the disease. Fresh litchi fruits were kept at −20°C until processing and analysis.

### 2.2 Winemaking processes

The method of Lee et al. (2018) was used with some modifications. 70 kg of raw materials was divided, peeled, destemmed, and crushed, and then all placed in 20‐L stainless steel tanks, and 60 mg/L sulfur dioxide was added for preservation. Physicochemical characteristics of litchi juice were as follows: total sugar, 125 g/L; titratable acidity (expressed as tartaric acid), 2.5 g/L. Afterward, the modified litchi juice was added with 20 mg/L pectinase and then kept at 5°C for 24 hr for clarification. As a starter, commercial yeast (*Saccharomyces cerevisiae* EC‐1118, Chr.‐Han, Horsholm, Denmark) 200 mg/L activated in advance (hydration in 5% of the glucose solution at 37°C for 30 min). The starter was added into the clear juice to start fermentation. And alcoholic fermentation was maintained at 25°C. Cap punching was performed three times a day during fermentation. When the residual sugar was lower than 2 g/L, the wines were centrifuged at 1,500 × g for 20 min, and the supernatants were transferred to the clean jars, treated with 60 mg/L sulfur dioxide, and stored at 4°C for six months. During the storage, a cold treatment at −4°C for 15 days was used to stabilize, and three times general racking was performed to clarify.

The distilled spirit was gained by alcoholic distillation with some modifications (Paolo et al., 1997). 200 ml of litchi wine, at 12% alcohol (v/v), was distilled in a copper distiller (Hoga Co., Salvaterra de Mino, Spain). A fraction of 50 ml at 43.9% alcohol (v/v) was collected. Four of these fractions were redistilled together, and a second 50 ml fraction at 83% alcohol was collected. The body of distillation was selected for testing; samples were recorded as distilled spirit.

Samples were collected at three different phases: raw juice, litchi wine, and litchi distilled spirit. Each sample was made in triplicate.

### 2.3 Chemicals and reagents

Analytical chemicals included tartaric acid, sodium chloride, anhydrous ethanol, and other reagents (Xi'an Chemical Industry Co. Ltd., China). The internal standard and chromatographically pure standards included ethyl caprylate, ethyl acetate, ethyl caprate, ethyl butyrate, isobutyl acetate, phenethyl acetate, diethyl succinate, ethyl laurate, octanoic acid, 1‐propanol, benzyl alcohol, phenethyl alcohol, 3‐hexen‐1‐ol, 2,3‐butanediol, isobutyric acid, isovaleric acid, phenylacetic acid, benzaldehyde, phenylacetaldehyde, geraniol, linalool, citronellol, 4‐terpineol, and 3,4‐dimethyl phenol. All volatile standards used for identification and quantification in this study were HPLC quality or GC grade, and their purities and commercial sources are listed in Table 1.

**TABLE 1:** Manufacturers, purities, quantitative ions, and emendation factors to 2‐octanol of the volatile standards used for the characterization of litchi wine and distilled spirit

No.	Compounds	Manufacturers^a^	Purity	Quantitative ions	Emendation factor A		
					1%	11%	28%
1	Ethyl caprylate	Sigma	99%	43 + 61+45	13.632	10.185	15.445
2	Ethyl acetate	Sigma	98%	RIC‐(35–400)	4.222	8.291	7.260
3	Ethyl caprate	Sigma	98%	RIC‐(35–400)	1.114	1.287	1.576
4	Ethyl butyrate	Sigma	98%	RIC‐(35–400)	1.385	0.998	0.786
5	Isobutyl acetate	Sigma	97%	RIC‐(35–400)	1.230	1.578	1.923
6	Phenethyl acetate	Aldrich	98%	RIC‐(35–400)	2.114	3.927	4.407
7	Diethyl succinate	Sigma	98%	101 + 29+129	11.456	10.232	8.793
8	Ethyl laurate	Aldrich	97%	88 + 101+43	0.472	0.371	1.233
9	Octanoic acid	Aldrich	98%	RIC‐(35–400)	3.212	3.065	4.219
10	1‐Propanol	Sigma	98%	73 + 43	4.241	2.334	3.788
11	Benzyl alcohol	Aldrich	97%	68 + 59	3.221	6.882	5.327
12	Phenethyl alcohol	Aldrich	98%	RIC‐(35–400)	2.114	1.008	0.653
13	1‐Propanol	Sigma	99%	31 + 29+27	13.493	14.375	14.770
14	2‐Octanol	Sigma	98%	RIC‐(35–400)	1	1	1
15	2,3‐Butanediol	Aldrich	97%	45 + 29	6.782	8.325	10.376
16	cis−3‐Hexen−1‐ol	Aldrich	98%	67 + 41+39	10.845	14.397	15.436
17	4‐Terpineol	Aldrich	97%	71 + 111+93	1.556	1.247	2.117
18	Benzyl alcohol	Sigma	98%	RIC‐(35–400)	17.189	17.322	13.376
19	Phenyl ethanol	Aldrich	99%	RIC‐(35–400)	19.434	28.445	21.32
20	Benzaldehyde	Aldrich	97%	RIC‐(35–400)	0.492	0.755	1.331
21	Phenylacetaldehyde	Sigma	97%	RIC‐(35–400)	2.339	3.129	3.785
22	Geraniol	Sigma	98%	94 + 129+112	4.120	4.313	8.998
23	Linalool	Sigma	98%	93 + 121+136	1.398	2.287	3.221
24	Citronellol	Aldrich	98%	93 + 124+112	0.768	2.834	2.137
25	3,4‐Dimethyl phenol	Aldrich	97%	RIC‐(35–400)	0.435	0.821	1.211

Note

A: Emendation factors to 2‐octanol (internal standard) of each volatile standard were measured in litchi wine and litchi distilled spirit containing 1%, 11%, and 28% (v/v) ethanol separately.

^a^ Manufacturers: Sigma‐Aldrich, St. Louis, MO, USA; Aldrich, Milwaukee, WI.

### 2.4 Volatile compounds analysis

The volatile compounds of the samples were isolated by stir bar sportive extraction (SBSE) according to the modified method proposed by Aguirre et al. (2014), and the volatile compounds of the samples were analyzed by GC‐MS as previously described by Lin et al. (2019).

#### 2.4.1 Extraction of volatile compounds

About 10 ml of each sample was placed in a 15‐ml airtight vial containing a magnetic stirrer bar (SBSE, 20 mm × 0.5 mm, Gerstel Co. Ltd, Germany), including 50 µl of 2‐octanol (0.234 mg/ml water, internal standard) and 2 g NaCl. Then, the airtight vial was placed on a magnetic stirrer (PC‐420D, Corning Co. Ltd, USA) and extracted for 90 min at 40°C with stirring (1,100 rpm). After the extraction, the stir bar was taken out with tweezers, washed it until there is no residual sample with distilled water, and dried with filter paper. Finally, it was inserted into the GC injector for 3 min to desorb analytes. The desorption temperature is 250°C. Each sample was extracted in triplicate.

#### 2.4.2 GC‐MS analysis

The separation, detection, and quantification of volatile compounds were performed on an Agilent TRACE GC (Agilent Technologies Inc., Santa Clara, CA, USA) coupled with Xcalibur V.3.0 mass spectrometry system (Thermo Fisher Scientific, Waltham, MA) and equipped with a DB‐WAXetr capillary column (30 m × 0.25 mm inner diameter, 0.25 μm film thickness, Agilent Technologies, USA). The conditions of GC‐MS in this study were applied as previously reported with some modifications (Yang et al., 2019). Ultrapure helium was used as the carrier gas at 1.0 ml/min. The initial column temperature was set at 40°C for 3 min. Afterward, the temperature was raised to 160°C at a rate of 5°C/min, then 7°C/min to 230°C, and held at 230°C for 8 min. Mass detector conditions were as follows: voltage of electron impact was 70 eV, scan range was m/z 30–350, and scanning frequency in full scan mode was 5.27 times/s.

#### 2.4.3 Quantification analysis of volatile compounds

Volatiles were identified by matching the obtained mass spectra with the Wiley libraries and by comparing the retention indices (RI) to those of the compounds reported in the NIST 2.0 and the literature. According to the method proposed by Wu et al. (2016), the quantification procedure was carried out with the internal standard quantification method with light modification. 2‐Octanol was employed as the internal standard compound. Three synthetic matrixes with 1%, 11%, and 28% (v/v) ethanol were prepared in distilled water, each contained 7.0 g/L tartaric acid, and the pH was adjusted to 3.6 with NaOH. Volatile standards were then extracted and analyzed under the same conditions as litchi juice, litchi wine, and distilled spirit samples to obtain their internal standard emendation factors. They are listed in Table 1. The emendation factors in the 1% standard solution were used to quantify volatile compounds in litchi juice. Those obtained in the 11% and 28% standard solution were used for volatile compounds in litchi wine and distilled spirit samples. The concentration of volatile compounds for which there was no pure reference available was estimated using the same emendation factor (obtained in the same standard solution) as one of the compounds with the most similar chemical structure.

#### 2.4.4 Determination of odor activity values

Odor activity value (OAV) is a parameter frequently used to assess each volatile compound's contribution to wine's aroma. Compounds existing in greater concentrations than their odor threshold (OAVs >1) are defined as aroma impact compounds and contribute individually to the wine aroma (Zhu et al., 2014). The influence of each aromatic compound on the scent of litchi wine and distilled spirit was determined by the odor activity values (OAVs), which were calculated as the ratio between the concentration and the odor threshold of the individual aroma compounds (Velázquez et al., 2015).

### 2.5 Statistical analysis

The data were reported as means ± standard deviation of three triplicates and were analyzed statistically by one‐way ANOVA. Means were compared by Duncan's multiple range test. Differences with p values <0.05 were considered statistically significant. The statistical software utilized was SPSS 17.0 (SPSS Inc., Chicago, IL, USA). To highlight the similarities and differences between wine samples and volatile compounds, principal component analysis (PCA) was carried out on the analytical data.

## 3 RESULTS AND DISCUSSION

### 3.1 Esters

**FIGURE 1:**
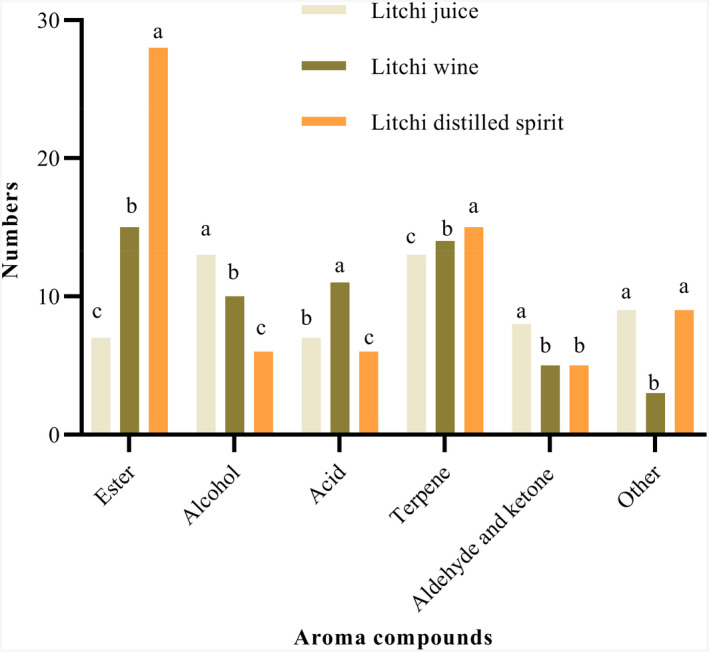
Comparison of the numbers of aroma compounds in litchi juice, wine, and distilled spirit. Values in the same column with different superscripted alphabet are significantly different at *p* < .05

**FIGURE 2:**
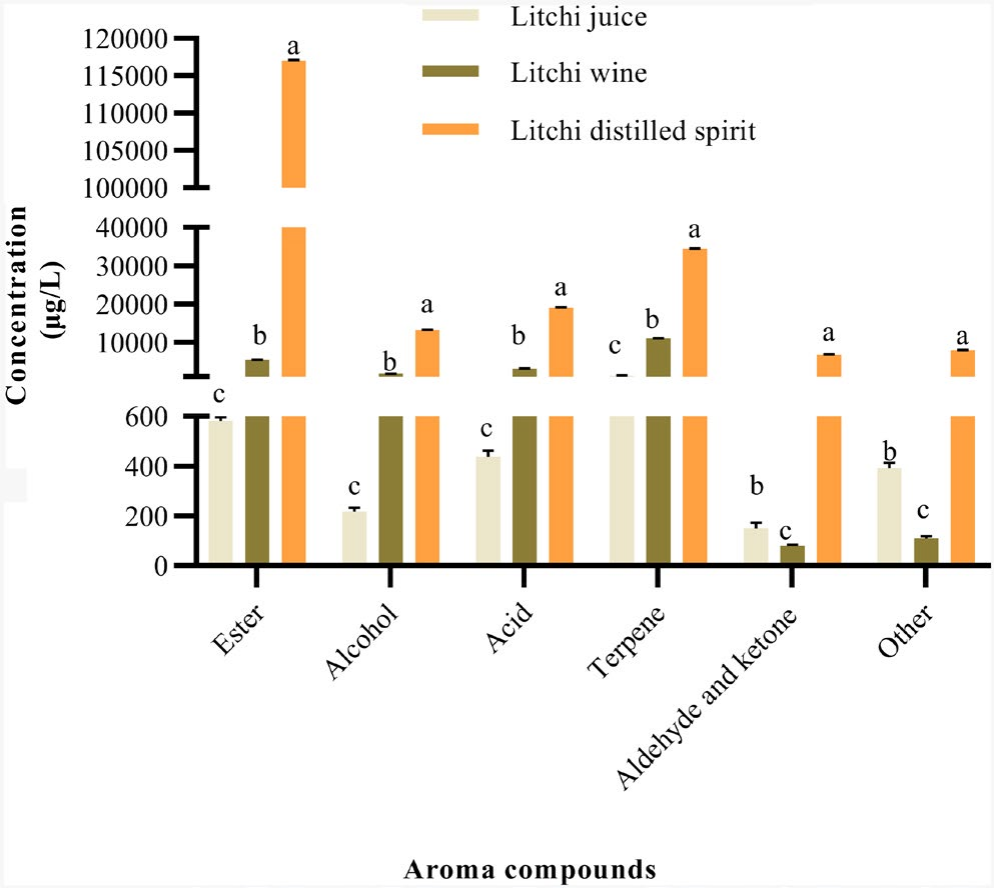
Comparison of the concentration (μg/L) of aroma compounds in litchi juice, wine, and distilled spirit. Values in the same column with different superscripted alphabet are significantly different at *p* < .05

Esters are of primary industrial interest because they have very low thresholds and can directly affect wine flavor and via complex synergistic interactions (Dzialo et al., 2017; Lytra et al., 2016). In this study, esters were the largest group in terms of the composition and concentration of aroma compounds. The esters' concentration ranged from 581.06 (litchi juice) to 117,121.99 µg/L (litchi distilled spirit), which was the highest among all six categories and represented approximately 52% of the total flavor component content (Figure 2). Similarly, Zhang et al. (2019) reported that esters represented about 40% of the total flavor component content (w/w) in hulless barley wine. Thirty‐nine different types of esters were identified (Table 2). The most inadequate variety of esters was observed in litchi juice; there were only seven esters, but 15 and 28 kinds of esters in litchi wine and distilled spirit (Figure 1), respectively. All esters those detected in litchi juices showed the increasing trend in the fermented ones except diisopropyl adipate, diethyl phthalate, and dibutyl phthalate (Table 2). In general, these compounds had a similar trend with a sharp increase during the winemaking and distilling process. Since esters are one of the most important by‐products of alcoholic fermentation and mainly produced by yeast as secondary products of sugar metabolism (Zhang et al., 2019). Our research also illustrates that 30 of 39 esters were newly generated during the winemaking process. Seven esters were just detected in litchi wine, including isoamyl acetate, isobutyl acetate, ethyl valerate, citronellyl acetate, diethyl succinate, ethyl 9‐decenoate, and 3‐hydroxytridecanoate ethyl. Some of these esters have also been found to be dominant in strawberry wine (Kafkas et al., 2006) and orange wine (Selli, 2007). These esters faded away in litchi distilled spirit. Conversely, another five esters (ethyl butyrate, ethyl caproate, ethyl caprylate, ethyl caprate, and ethyl palmitate) increased sharply at the end of the distilling process. Ethyl acetate (1973.52 ± 142.78 µg/L), ethyl caprylate (1,296.28 ± 110.93 µg/L), and isoamyl acetate (545.70 ± 45.02 µg/L) were the most abundant esters in litchi wine. Similarly, Ayestarán et al. (2019) also found the highest isoamyl acetate levels in Tempranillo Blanco wines. These aroma compounds might be significant contributors to the characteristic aroma of litchi wine. In the finished litchi distilled spirit, ethyl palmitate was most abundant among esters and represented approximately 65% of the total ester content (256.2 mg/L), followed by ethyl linoleate (19,815.3 ± 1,232.1 µg/L), ethyl linolenate (15,981.76 ± 32.00 µg/L), and ethyl caprylate (14,288.9 ± 737.72 µg/L). In particular, the increase in ethyl acetate concentration in litchi wine and especially in litchi distilled spirit with respect to litchi juice was possibly caused by the action of *Saccharomyces cerevisiae* (Erasmus et al., 2004), as it was emphasized by another author who found a similar trend in durian pulp wine (Lu et al., 2017). Based on the odor activity values (Table 3), 12 types of esters showed OAVs >1 in three samples. Litchi wine presented a total of 6 esters with OAVs >1. Among them, ethyl caproate (OAV of 29.62), ethyl caprylate (OAV of 5.18), and ethyl butyrate (OAV of 4.47) were presented with relatively high OAVs and might be significant contributors to the aroma of litchi wine. However, these esters' OAVs increased sharply in distilled spirit except isoamyl acetate and were 5 to 11 times higher in distilled spirit than in litchi wine. Additionally, five types of new esters were presented in distilled spirit with OAVs >1, including cis‐whiskey lactone (OAV of 4.46), citronellyl acetate (OAV of 4.2), ethyl laurate (OAV of 5.07), ethyl myristate (OAV of 1.81), ethyl palmitate (OAV of 1.81). It is noteworthy that the OAVs of ethyl butyrate (OAV of 22), ethyl isovalerate (OAV of 16.6), and ethyl caproate (OAV of 206) were much higher than its odor thresholds in the distilled spirit. These aroma compounds might explain the fruit aroma in the distilled spirit.

**TABLE 2:** Concentration (μg/L) of volatile compounds detected in litchi juice, litchi wine, and distilled spirit

Code	Aroma compound	Concentration (μg/L)		
		Litchi juice	Litchi wine	Litchi distilled spirit
	*Esters*			
E1	Ethyl acetate	49.52 ± 7.21c	1973.52 ± 142.78b	3,933.12 ± 267.09a
E2	Diisopropyl adipate	81.21 ± 10.54a	nd	nd
E3	cis‐Whiskey lactone	nd	nd	298.72 ± 42.22a
E4	Isobutyl acetate	nd	91.67 ± 9.54a	nd
E5	Ethyl butyrate	nd	89.49 ± 5.09b	448.08 ± 52.34a
E6	Ethyl isovalerate	nd	8.73 ± 0.65b	49.78 ± 4.87a
E7	Isoamyl pyruvate	nd	nd	1,294.46 ± 111.02a
E8	linalyl caproate	nd	nd	846.38 ± 68.77a
E9	5‐Oxohexanethioic acid,s‐t‐butyl ester	nd	nd	1,254.26 ± 143.09a
E10	Isoamyl acetate	nd	545.70 ± 45.02a	nd
E11	Ethyl valerate	nd	2.18 ± 0.03a	nd
E12	Ethyl caproate	nd	414.72 ± 36.82b	2,887.64 ± 276.34a
E13	Ethyl caprylate	nd	1,296.28 ± 110.93b	14,288.9 ± 737.72a
E14	Terpinyl formate	nd	nd	746.80 ± 87.57a
E15	Ethyl caprate	nd	408.18 ± 38.78b	13,293.2 ± 462.38a
E16	Citronellyl acetate	nd	185.54 ± 20.13a	nd
E17	Diethyl succinate	nd	43.66 ± 5.11a	nd
E18	Ethyl 9‐decenoate	nd	54.57 ± 4.37a	nd
E19	Ethyl−3‐hydroxytridecanoate	nd	34.92 ± 2.13a	nd
E20	Terpinyl formate	nd	nd	746.80 ± 87.57a
E21	Geranyl isovalerate	40.28 ± 5.11b	nd	799.08 ± 50.89a
E22	Citronellyl acetate	nd	nd	2,688.5 ± 21.42a
E23	Ethyltrans−4‐decenoate	nd	nd	2,688.5 ± 307.34a
E24	Ethyl laurate	nd	nd	497.87 ± 34.87a
E25	Ethyl myristate	nd	nd	7,617.44 ± 568.32a
E26	Methyl Z−11‐tetradecenoate	nd	nd	3,634.46 ± 643.99a
E27	Pentadecanoic acid, ethyl ester	nd	nd	647.24 ± 58.44a
E28	Methyl 14‐methylpentadecanoate	nd	nd	1513.41 ± 20.90a
E29	Ethyl 9‐hexadecenoate	nd	nd	6,771.06 ± 703.28a
E30	4‐Nitrophenylhexadecanoate	nd	nd	5,327.24 ± 92.11a
E31	Ethyl palmitate	nd	117.87 ± 5.39b	75,880.12 ± 123.2a
E32	Ethyl 15‐methylheptadecanoate	nd	nd	6,024.90 ± 78.89a
E33	Ethyl oleate	nd	nd	63.66 ± 5.92a
E34	Ethyl linoleate	nd	nd	19,815.3 ± 1,232.1a
E35	Ethyl linolenate	nd	nd	15,981.76 ± 32.00a
E36	Dimethyl phthalate	80.87 ± 9.08b	102.53 ± 7.43a	nd
E37	Diethyl phthalate	91.67 ± 9.23a	nd	nd
E38	Diisobutyl phthalate	182.59 ± 21.22b	nd	2,539.14 ± 347.23a
E39	Dibutyl phthalate	54.92 ± 8.34a	nd	nd
	Alcohols			
A1	2‐Methyl−3‐buten−2‐ol	17.83 ± 1.79b	nd	297.37 ± 36.04a
A2	1‐Pentanol	30.37 ± 2.46c	763.98 ± 48.90b	4,132.34 ± 320.90a
A3	Isoamyl alcohol	nd	255.39 ± 28.31b	2,837.88 ± 247.28a
A4	3‐Methyl−3‐buten−1‐ol	9.90 ± 0.08b	nd	149.36 ± 28.47a
A5	Hexyl alcohol	15.85 ± 1.78a	2.18 ± 0.07b	nd
A6	3‐Octanol	nd	85.13 ± 6.09b	248.94 ± 18.99a
A7	1‐Octen−3‐ol	52.29±0.4.38a	56.75 ± 7.26a	nd
A8	2‐Ethylhexanol	23.11 ± 0.38b	37.11 ± 4.31a	nd
A9	3‐Methylthiopropanol	52.29±0.4.38a	45.84 ± 2.80a	nd
A10	7‐Methyl−3‐methylene−6‐Octen−1‐ol	nd	82.95 ± 3.42a	nd
A11	2‐(4‐methylene‐cyclohexyl)−2‐propen−1‐ol	23.11 ± 0.38b	61.12 ± 7.00a	nd
A12	Phenethyl alcohol	nd	464.94 ± 78.26a	nd
A13	trans‐p−2,8‐Menthadien−1‐ol	27.78 ± 4.39a	nd	nd
A14	Furfuryl alcohol	22.97 ± 2.08a	nd	nd
A15	2,7‐Octadien−4‐ol,2‐methyl−6‐methylene‐	27.28 ± 1.19a	nd	nd
A16	7‐Methyl−3‐methylene−6‐octen−1‐ol	6.94 ± 0.03c	82.95 ± 4.56b	5,476.75 ± 10.23a
A17	2,6‐Octadiene−1,8‐diol,2,6‐dimethyl‐	27.24 ± 3.24a	nd	nd
	*Acids*			
AC1	2‐Ethylexanoic acid	49.68 ± 3.87a	nd	nd
AC2	Ethylboronic acid	30.98 ± 4.32c	63.30 ± 3.19b	497.86 ± 66.32a
AC3	Methylenecyclopropane−2‐carboxylic acid	nd	13.10 ± 0.09a	nd
AC4	Acetic acid	nd	96.04 ± 7.23a	nd
AC5	Hexanoic acid	71.04 ± 9.46a	52.38 ± 4.90b	nd
AC6	Octanoic acid	138.36 ± 14.68c	964.80 ± 81.21b	3,335.74 ± 289.07a
AC7	Nonanoic acid	63.04 ± 6.54a	nd	nd
AC8	3,7‐dimethyl−6‐Octenoic acid	nd	189.90 ± 34.78a	nd
AC9	Decanoic acid	50.75 ± 6.89c	993.17 ± 100.78b	3,476.10 ± 198.33a
AC10	3,7‐dimethyl−6‐octadienoic acid	nd	373.26 ± 45.87a	nd
AC11	Lauric acid	nd	106.96 ± 11.34a	nd
AC12	Myristic acid	nd	133.15 ± 18.35b	3,485.10 ± 378.62a
AC13	Linoleic acid	nd	91.68 ± 5.32a	nd
AC14	Pentadecanoic acid	nd	nd	3,026.80 ± 276.55a
AC15	14‐Pentadecenoic acid	nd	nd	1,045.52 ± 87.22a
AC16	Diethylenetriaminepentaacetic acid	42.74 ± 3.98a	nd	nd
	*Terpenes*			
T1	cis‐Rose oxide	169.34 ± 18.42c	635.19 ± 48.09b	5,053.40 ± 48.88a
T2	trans‐Rose oxide	75.32 ± 7.21c	279.40 ± 34.21b	1867.02 ± 198.34a
T3	(1S)‐(1)‐beta‐Pinene	nd	6.54 ± 0.98b	127.56 ± 14.08a
T4	D‐Sylvestrene	nd	8.73 ± 0.34b	248.94 ± 16.87a
T5	Terpinolene	nd	10.91 ± 0.79a	nd
T6	p‐Mentha−3,8‐diene	nd	nd	199.14 ± 21.22a
T7	2,6‐Dimethyl−1,3,5,7‐octatetrene	nd	nd	297.86 ± 31.24a
T8	5‐Caranol	21.37 ± 2.91c	nd	2,788.08 ± 300.90a
T9	b‐Guaiene	nd	nd	995.74 ± 120.38a
T10	Carveol	8.03 ± 0.05b	54.57 ± 8.39a	‐
T11	Linalool	85.18 ± 8.25c	772.71 ± 37.77b	4,381.28 ± 381.34a
T12	4‐Terpineol	28.84 ± 1.19a	nd	nd
T13	p‐Menth−1‐en−8‐ol	71.58 ± 6.32b	1672.02 ± 129.02a	nd
T14	cis‐Carveol	33.65 ± 3.13a	nd	nd
T15	D‐Citronellol	263.89 ± 30.67c	5,662.18 ± 438.70b	8,488.72 ± 789.32a
T16	cis‐Geraniol	33.12 ± 3.12c	528.24 ± 36.90b	1,344.26 ± 78.99a
T17	trans‐Carveol	nd	91.68 ± 8.73a	nd
T18	Neroloxide	nd	67.67 ± 8.32b	2,937.44 ± 264.19a
T19	iso‐Geraniol	50.21 ± 6.51c	870.74 ± 76.32b	3,534.88 ± 200.15a
T20	Geraniol	397.44 ± 46.88c	870.74 ± 48.77b	1593.22 ± 07.32a
T21	Elemicin	87.07 ± 4.32a	nd	nd
T22	cis‐Nerolidol	nd	nd	597.44 ± 48.41a
	*Aldehydes and ketones*			
AK1	Hexanal	18.69 ± 3.21a	nd	nd
AK2	Acetal	nd	nd	2,489.36 ± 235.21a
AK3	trans−2‐Hexenal	10.56 ± 1.99a	nd	nd
AK4	2‐Isopropylidene−5‐methyhex−4‐enal	20.83 ± 3.01a	nd	nd
AK5	3,7‐dimethylnona−2,6‐dienal	nd	10.91 ± 0.29a	nd
AK6	3‐Furaldehyde	nd	41.47 ± 7.21a	nd
AK7	1,3‐Dioxolane−4‐methanol	nd	4.58 ± 0.92a	nd
AK8	2‐Methyl−3‐octanone	nd	8.73 ± 0.89a	nd
AK9	2‐Octanone	20.84 ± 2.00a	10.91 ± 0.77b	nd
AK10	Acetophenone	27.07 ± 1.54a	nd	nd
AK11	Cyclohexanone, 2‐cyclohexyl‐	nd	nd	99.58 ± 10.32a
AK12	2‐Methyltetrahydrothiophen−3‐one	nd	nd	398.28 ± 28.45a
AK13	3,6,6‐Trimethylundecane−2,5,10‐trione	nd	nd	3,186.38 ± 273.89a
AK14	β‐Damascenone	nd	nd	647.24 ± 77.46a
AK15	3‐Isopropylidene−5‐methyhex−4‐en−2‐one	7.25 ± 0.08a	nd	nd
AK16	1‐Butanone,1‐(2,4,6‐trihydroxyphenyl)	49.24 ± 2.87a	nd	nd
AK17	6‐Methyl−5‐hepten−2‐one	22.45 ± 2.58a	nd	nd
	Others			
S1	2,6‐methyl−2‐octene	176.94 ± 12.35a	nd	nd
S2	Durenol	33.68 ± 1.90a	nd	nd
S3	1,4‐Benzenediol, dimethyl‐	18.70 ± 0.05a	nd	nd
S4	2,4,6‐Triisopropylphenol	43.27 ± 2.42a	nd	nd
S5	4,6‐Di‐tert‐butyl−2‐methylphenol	46.87 ± 3.78a	nd	nd
S6	Pentylcyclopropane	48.61 ± 4.21	nd	nd
S7	1,1‐Diethoxy−2‐methylbutane	nd	4.37 ± 0.48a	53.44 ± 7.45a
S8	1,1‐Diethoxy−3‐methylbutane	nd	nd	99.58 ± 6.31a
S9	1‐(1‐Ethoxyethoxy)‐pentane	nd	4.39 ± 0.92a	298.72 ± 33.28a
S10	2‐Pentylfuran	13.21 ± 0.09a	8.73 ± 0.75a	34.85 ± 5.32a
S11	Trimethylhydrazine	26.18 ± 3.23a	nd	nd
S12	Mesylazide	9.90 ± 4.11a	nd	nd
S13	2‐Ethyl‐p‐xylene	nd	nd	149.36 ± 17.23a
S14	Verbenyl ethyl ether	nd	nd	1692.76 ± 187.33a
S15	Styrene, 3,4‐dimethyl‐	nd	nd	1842.12 ± 88.89a
S16	Benzene,1,2,3,5‐tetramethyl‐	nd	nd	1593.18 ± 123.89a
S17	2,4‐di‐tert‐butylphenol	nd	nd	2041.26 ± 253.12a

Note

Data are expressed as mean ± standard deviation (*n* = 3). a‐c means with different lowercase letters in the same row indicate significant differences (*p* <.05);

“–”: Not detected.

**TABLE 3:** Odor description, odor threshold, and odor activity value of main volatile compounds found in litchi juice, litchi wine, and distilled spirit

Code	Volatile compound	Odor description^a^	Odor threshold (μg/L)^b^	Odor activity value		
				Litchi juice	Litchi wine	Distilled spirit
E1	Ethyl acetate	Pineapple	7,500	0.006	0.26	0.52
E3	cis‐Whiskey lactone	Fruit, cocoa	6	0.0	0.0	4.46
E4	Isobutyl acetate	Fruit, apple, banana	1,600	0.0	0.06	0.0
E5	Ethyl butyrate	Apple, banana	20	0.0	4.47	22
E6	Ethyl isovalerate	Fruit	3	0.0	2.91	16.6
E10	Isoamyl acetate	Banana	200	0.0	2.72	0.0
E12	Ethyl caproate	Apple	14	0.0	29.62	206
E13	Ethyl caprylate	Sweet	250	0.0	5.18	57.15
E15	Ethyl caprate	Pineapple, flower	200	0.0	2.04	66
E17	Diethyl succinate	Wine, fruit	1,200	0.0	0.04	0.0
E18	Ethyl 9‐decenoate	Fat	100	0.0	0.55	0.0
E22	Citronellyl acetate	Pineapple, flower	640	0.0	0.3	4.2
E24	Ethyl laurate	Leaf	1,500	0.0	0.0	5.07
E25	Ethyl myristate	Ether	2000	0.0	0.0	1.81
E31	Ethyl palmitate	Apple, sweet	1,500	0.0	0.08	50.59
A2	1‐Pentanol	Alcohol, pungent	80,000	0.0003	0.009	0.05
A3	Isoamyl alcohol	Wine, solvent, bitter	7,000	0.0	0.04	0.4
A4	3‐Methyl−3‐butene−1‐ol	Apple	600	0.02	0.0	0.2
A5	Hexyl alcohol	Resin, flower, green	8,000	0.002	0.0002	0.0
A7	1‐Octen−3‐ol	Mushroom	18	2.91	3.15	0.0
A8	2‐Ethylhexanol	Rose, green	900	0.03	0.04	0.0
A9	3‐Methylthiopropanol	Sweet potato	1,000	0.05	0.05	0.0
A11	Phenethyl alcohol	Rose	1,100	0.0	0.42	0.0
AC4	Acetic acid glacial	Vinegar	200 mg/L	0.0	0.0005	0.0
AC5	Hexanoic acid	Barbecue, cheese	420	0.2	0.12	0.0
AC6	Octanoic acid	Sweat, cheese	500	0.14	1.93	6.7
AC9	Decanoic acid	Rancid, fat	1,000	0.05	1	3.5
AC11	Lauric acid	Metal	1,500	0.0	0.07	0.0
T1	cis‐Rose oxide	Rose, flower	20	8.5	3.2	252.67
T2	trans‐Rose oxide	Rose, flower	20	3.8	1.4	93.36
T11	Linalool	Floral	15	5.7	51.51	292.09
T12	4‐Terpineol	Turpentine, nutmeg, must	110	0.26	0.0	0.0
T13	p‐Menth−1‐en−8‐ol	Fruit, flower	250	0.3	6.68	0.0
T15	D‐Citronellol	Clove	100	2.63	56.62	84.88
T16	cis‐Geraniol	Rose	300	0.11	1.76	4.48
T18	Neroloxide	Rose	6,000	0.0	0.01	0.49
T20	Geraniol	Rose	130	3.1	3.44	12.25
AK9	2‐Octanone	Hot milk, peanut, green	250	0.0	0.04	0.0
AK14	β‐Damascenone	Apple	0.5	0.0	0.0	1,294.48

^a^ http://www.flavornet.org/flavornet.html.

^b^ Odor threshold (μg/L) reported in the literature (Feng et al., 2018; Guth, 1997; Lukić et al., 2016; Wu et al., 2011).

### 3.2 Alcohol

Alcohols, also called ‘fusel alcohols’, which are the main group of volatile metabolites synthesized by yeast during alcoholic fermentation, whereas only tiny quantities derive from the litchi (Dzialo et al., 2017; Ma et al., 2017). The greatest amount of alcohols was observed in the distilled spirit, in which the variety of alcohols was actually the lowest (Figure 1 and Figure 2). Conversely, the litchi juice showed the greatest variety with a minimum of alcohols concentration. Four alcohols were just detected in litchi juice (Table 2), namely trans‐p‐2,8‐menth‐adien‐1‐ol (27.78 ± 4.39 µg/L), 2,6‐octadiene‐1,8‐diol,2,6‐dimethyl‐ (27.24 ± 3.24 µg/L), 2,7‐octadien‐4‐ol,2‐methyl‐6‐methylene‐ (27.78 ± 4.39 µg/L), and furfuryl alcohol (22.97 ± 2.08 µg/L). These alcohols faded away during alcoholic fermentation. Another 2 alcohols, hexyl alcohol and 3‐methylthiopropanol, had a higher concentration in litchi juice but decreased and were only detected in litchi wine. Similar to previous results reported by Feng et al. (2018), who also observed hexyl alcohol in raw Sweetheart lychee. On the contrary, alcohols, such as 1‐pentanol, 1‐octen‐3‐ol, 2‐ethylhexanol, 2‐(4‐methylene‐cyclohexyl)‐2‐propen‐1‐ol, 7‐methyl‐3‐methylene‐6‐octen‐1‐ol, increased along with the fermentation. The amounts of the major alcohols 1‐pentanol and isoamyl alcohol and 3‐octanol increased in the distilled spirit in relation to litchi wine, what is similar to results reported by Chen and Liu (2016), who also reported the higher isoamyl alcohol in litchi (*Litchi chinensis* Sonn. var. Nuomi Ci) wine. The highest concentration of 7‐methyl‐3‐methylene‐6‐octen‐1‐ol (5,476.75 ± 10.23 µg/L) was found in litchi distilled spirit, followed by 1‐pentanol (4,132.34 ± 320.90 µg/L) and isoamyl alcohol (2,837.88 ± 247.28 µg/L). 1‐octen‐3‐ol presenting OAV >1 was only found in litchi wine (Table 3), which is different from the study of Tang et al. (2019), who did not find 1‐octen‐3‐ol in litchi (Huaizhi) wine.

### 3.3 Acids

Some acids in litchi distilled spirit are naturally present in litchi juice, while others are by‐products of fermentation (Venkatachalam et al., 2018). This group of volatiles could actively contribute to wine flavor at low levels. The widest variety of acids was observed in litchi wine, and there were 11 kinds of acids, but 7 and 6 kinds of acids in litchi juice and litchi distilled spirit, respectively. The concentration of total acids ranged from 446.59 (litchi juice) to 19058.24 µg/L (litchi distilled spirit) (Figure 2). Higher concentrations of octanoic acid (964.80 ± 81.21 µg/L) and decanoic acid (993.17 ± 100.78 µg/L) were recorded in litchi wine (Table 2); these values were higher than the values reported previously in lychee wine (319.58 ± 9.33 µg/L and 56.74 ± 0.87 µg/L) (Tang et al., 2019), but they were much lower than the amount at the end of alcohol fermentation of litchi wine (8,339.0 ± 794.2 µg/L and 10,711.9 ± 3,499.5 µg/L) (Wu et al., 2011). The apparent discrepancy may reflect differences in the type of raw litchi or the fermentation process's duration. Myristic acid (3,485.10 ± 378.62 µg/L), decanoic acid (3,476.10 ± 198.33 µg/L), octanoic acid (3,335.74 ± 289.07 µg/L), and pentadecanoic acid (3,026.80 ± 276.55 µg/L) were presented in higher concentration in distilled spirit. Only two types of acids showed OAVs >1 in our study (Table 3), namely decanoic acid and octanoic acid. The OAVs of octanoic acid and decanoic acid increased sharply during the distilled process. They were 3.5 times higher in distilled spirit than in litchi wine, consistent with the trends of their concentrations. However, unpopular sweat and rancid aromas might not be generated in distilled spirit because the OAVs of octanoic acid (OAV of 6.7) and decanoic acid (OAV of 3.5) were much lower than its odor thresholds.

### 3.4 Terpenes

Terpenes, synthesized from glucose by the isoprenoid pathway, provide a powerful floral and fruity aroma to berries (Venkatachalam et al., 2018; Wu et al., 2016). In the present study, 22 terpenes were detected in three treatments (Table 2). The kinds of total aroma compounds did not differ significantly among the three treatments (Figure 1). In contrast, the total contents of aroma compounds significantly differed among these treatments. Thirteen terpenes were found in litchi juice, namely cis‐rose oxide (169.34 ± 18.42 µg/L), trans‐rose oxide (75.32 ± 7.21 µg/L), 5‐caranol (21.37 ± 2.91 µg/L), carveol (8.03 ± 0.05 µg/L), linalool (85.18 ± 8.25 µg/L), 4‐terpineol (28.84 ± 1.19 µg/L), p‐menth‐1‐en‐8‐ol (71.58 ± 6.32 µg/L), cis‐carveol (33.65 ± 3.13 µg/L), D‐citronellol (263.89 ± 30.67 µg/L), cis‐geraniol (33.12 ± 3.12 µg/L), iso‐geraniol (50.21 ± 6.51 µg/L), geraniol (397.44 ± 46.88 µg/L), and elemicin (87.07 ± 4.32 µg/L) (Table 2). Except for 4‐terpineol, cis‐carveol, and elemicin, which were typically present in litchi juice but faded away in litchi wine and distilled spirit, these terpenes were sharply increased during the whole winemaking and distilling process. Geraniol, linalool, and cis‐rose oxide were higher than other terpenes in litchi juice; this trend was similar to the results reported by Chen and Liu (2016) in litchi juice. Geraniol could be metabolized into cis‐rose oxide by some yeast strains (Steyer et al., 2012). Simultaneously, cis‐rose oxide had a positive effect on the rose aroma of wine because of its high odor activity (Guth, 1997). Linalool is produced by many flowers and spice plants with a pleasant floral scent and can also contribute sweet and citrus scents to the overall aroma profile of litchi. Among these various terpenes, D‐citronellol (5,662.18 ± 438.70 µg/L) and p‐menth‐1‐en‐8‐ol (1672.02 ± 129.02 µg/L) were predominant in litchi wine; in contrast, D‐citronellol (8,488.72 ± 789.32 µg/L), cis‐rose oxide (5,053.40 ± 48.88 µg/L), linalool (4,381.28 ± 381.34 µg/L), and iso‐Geraniol (3,534.88 ± 200.15 µg/L) were also predominant in litchi distilled spirit. These terpenes, including cis‐rose oxide, trans‐rose oxide, linalool, geraniol, citronellol, and carveol, were previously reported in litchi wine (Chen & Liu, 2016; Tang et al., 2019; Wu et al., 2011). A total of 7 and 6 terpenes were presented with OAVs higher than 1 in litchi juice and distilled spirit (Table 3), respectively. Among them, D‐citronellol (OAV of 56.62), linalool (OAV of 51.51), p‐menth‐1‐en‐8‐ol (OAV of 6.68), geraniol (OAV of 3.44), and cis‐rose oxide (OAV of 3.2) were presented with relatively high OAVs and might be important contributors to the aroma of litchi wine. Moreover, linalool (OAV of 292.09), cis‐rose oxide (OAV of 252.67), and trans‐rose oxide (OAV of 93.36) had the highest OAVs in distilled spirit, and the OAVs were much higher than its odor thresholds. These aroma compounds might explain the unique rose and flower aroma in the distilled spirit.

### 3.5 Aldehydes and ketones

Aldehydes and ketones could confer a more abundant, more elegant, and unique aroma to wine (Nyanga et al., 2013). The widest variety of aldehydes and ketones was observed in litchi juice. There were eight kinds of aldehydes and ketones, but 5 and 5 kinds of aldehydes ketones in litchi wine and litchi distilled spirit (Table 2 and Figure 1), respectively. Conversely, the highest total concentration of aldehydes and ketones was found in litchi distilled spirit (6,820.84 µg/L). The kinds of aroma compounds were absolutely different among the three treatments except for the 2‐octanone, which is detected in litchi juice and litchi wine with peanut and green aroma. Among the various aldehydes and ketones, 3‐furaldehyde (41.47 ± 7.21 µg/L), 3,7‐dimethylnona‐2,6‐dienal (10.91 ± 0.29 µg/L), and 2‐octanone (10.91 ± 0.77 µg/L) were predominant in litchi wine, while 3,6,6‐trimethylundecane‐2,5,10‐trione (3,186.38 ± 273.89 µg/L) and acetal (2,489.36 ± 235.21 µg/L) were predominant in distilled spirit, which represented approximately 83% of the total aldehydes and ketones content. β‐damascenone presented the most robust odor (OAV of 1,294.48), increased significantly during the distilling process, and was only found in the distilled spirit. Therefore, apple and plum odor could be expected in the distilled spirit, as suggested by Genovese et al. (2007) and Lukić et al. (2016), who observed a similar trend in wines made from overripe grapes.

### 3.6 Others

The variety and content of others in litchi wine were actually the lowest, while the total contents of others in litchi distilled spirit represented approximately 94%. Nine aroma compounds of others were identified in litchi juice. Among these aroma compounds, 2,6‐methyl‐2‐octene, 2,4,6‐triisopropylphenol, dureno, 1,4‐benzenediol, dimethyl‐, pentylcyclopropane, trimethylhydrazine, mesylazide were just found in litchi juice and fade away in litchi wine. In particular, 2‐pentylfuran was the only one and presented in the whole winemaking and distilling process. 2‐Pentylfuran was also determined in cv. Dimrit grape seed oil. These author also reported that presence of 2‐pentylfuran may be associated with the applied high temperature during the oil extraction procedure (Sevindik et al., 2020). Six aroma compounds were newly generated during the distilling process. In the finished litchi distilled spirit, 2,4‐di‐tert‐butylphenol (2041.26 ± 253.12 µg/L) was most abundant and represented approximately 26% of the total contents, followed by benzene,1,2,3,5‐tetramethyl‐(1593.18 ± 123.89 µg/L), styrene,3,4‐dimethyl‐ (1842.12 ± 88.89 µg/L), and verbenyl ethyl ether (1692.76 ± 187.33 µg/L).

### 3.7 Analysis of the characteristic volatiles by principal component analysis (PCA) with OAVs (>1)

**FIGURE 3:**
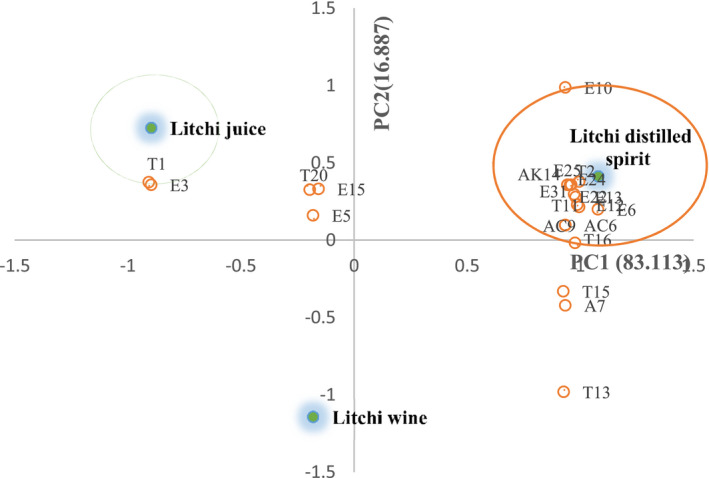
Principal components analysis for the main volatiles (OAVs >1) in litchi juice, wine, and distilled spirit

PCA was conducted to understand the correlation and segregation among those volatile compounds, which are significantly different among the three samples. Twenty‐two essential volatile compounds were selected for PCA to determine the contribution with OAVs of >1 in Table 3. The richest variety of OAVs >1 was observed in litchi distilled spirit; there were 19 kinds of volatile aroma compounds, but 6 and 16 kinds of aroma compounds in litchi juice and litchi wine, respectively. As shown in Figure 3, the three samples were differentiated by their main aroma profiles. The compound cis‐rose oxide (T1) was more related to litchi juice. Four kinds of volatile compounds may be related to litchi wine, including E5 (ethyl butyrate), T13 (p‐menth‐1‐en‐8‐ol), T15 (D‐citronellol), and A7 (1‐octen‐3‐ol). And six kinds of volatile compounds were more related to litchi distilled spirit, including E12 (ethyl caproate), E6 (ethyl isovalerate), T2 (trans‐rose oxide), T11 (linalool), and AK14 (β‐damascenone).

**FIGURE 4:**
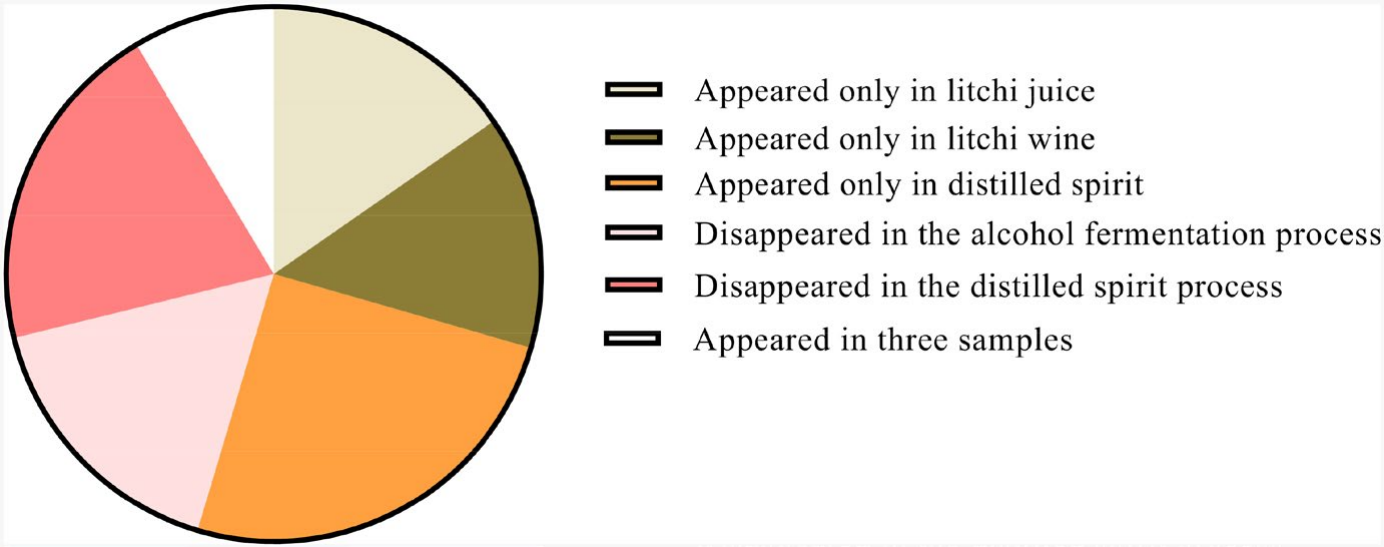
Changes of the numbers of aroma compounds in winemaking process

The kinds of total volatile aroma compounds appeared only in distilled spirit were highest (Figure 4 and Table 2), and there were 41 kinds of compounds in distilled spirit, but 25 and 23 in litchi juice and litchi wine, respectively. This illustrated fermentation and distilled spirit process lead to a series of by‐products. They include esters, alcohols, acids, terpenes, and so on, all of which influence the final wine's quality. The concentration of the by‐products can vary widely from a few g/L to hundreds of mg/L. Conversely, five kinds of volatile aroma compounds were disappeared in the alcohol fermentation process, and 33 disappeared in the distilled spirit process. Fourteen kinds of volatile aroma compounds detected in litchi juice showed increasing trends during alcoholic fermentation and distilled spirit process. Notably, some volatile aroma compounds detected in distilled spirit were ten times higher than litchi wine, such as ethyl caprylate (11.02), ethyl caprate (32.57), ethyl palmitate (643.76), isoamyl alcohol (11.11), 7‐methyl‐3‐methylene‐6‐octen‐1‐ol (66.02), myristic acid (26.17), (1S)‐(1)‐beta‐pinene (19.50), D‐sylvestrene (28.52), neroloxide (43.41), 1,1‐diethoxy‐2‐methylbutane (12.23) and 1‐(1‐ethoxyethoxy)‐pentane (68.05). According to the determined numbers of total aroma components (Figure 1), the proportion of esters in litchi distilled spirit was higher, while terpenes in litchi wine was higher, suggesting the difference of aroma in litchi wine and distilled spirit.

The presence of larger amounts of bioactive compounds in litchi assures its considerable nutritional value. Concentration (µg/L) of volatile compounds detected in litchi (Heiye and Guiwei) is shown in Table S1. 1‐Pentanol (30.37 µg/L), 3‐methyl‐3‐butene‐1‐ol (9.90 µg/L), 1‐octen‐3‐ol (52.29 µg/L), linalool (85.18 µg/L), 4‐terpineol (28.84 µg/L), trans‐2‐hexenal (18.56 µg/L) and 6‐methyl‐5‐hepten‐2‐one (22.45 µg/L) have higher concentration in litchi (Heiye) than litchi (Guiwei). Pieces of literature also indicate that litchi (Heiye) mainly includes higher total phenolics, total flavonoids, chlorogenic acid, and gallic acid than litchi (Guiwei) (Zhang et al., 2013; Zhao et al., 2020). Moreover, cis‐rose oxide, trans‐2‐hexenal, linalool, and geraniol had the highest OAVs in litchi (Heiye and Guiwei), much higher than its odor thresholds (Table S1). These aroma compounds might be important contributors to the unique aroma of litchi, agreeing with previous reports by Tang et al. (2019). They also observed higher OAVs of linalool (OAV of 43.270 ± 0.767), cis‐rose oxide (OAV of 99.969 ± 9.009), and geraniol (OAV of 1.866 ± 0.071) in litchi (Huaizhi). Notably, for the first time, the volatile aroma compounds in litchi distilled spirit (Heiye) were identified in this study. Compared with the Gewürztraminer wine, the concentrations of ethyl butyrate, cis‐rose oxide, linalool, and β‐damascenone were 403 ± 34 µg/L, 4.6 ± 0.8 µg/L, 35.9 ± 1.3 µg/L, and 2.0 ± 0.3 µg/L, respectively, and litchi distilled spirit possessed higher concentrations of ethyl butyrate (448.08 ± 52.34 µg/L), cis‐rose oxide (5,053.40 ± 48.88 µg/L), linalool (4,381.28 ± 381.34 µg/L), β‐damascenone (647.24 ± 77.46 µg/L) (Lukić et al., 2016). It is possible that the produced litchi distilled spirit had a stronger varietal character due to the increased concentrations and OAVs of β‐damascenone, linalool, ethyl isovalerate, ethyl butyrate, ethyl caproate, trans‐rose oxide, and cis‐rose oxide (Table 2 and Table 3) since these compounds are known to play a decisive fruit and flower aroma in the distilled spirit. Similar to previous results reported by Wu et al. (2011), who also observed higher OAVs of ethyl butyrate (OAV of 69.1 ± 4.0), cis‐rose oxide (OAV of 62.7 ± 0.1), and trans‐rose oxide (OAV of 20.6 ± 0.4) in litchi wine.

## 4 CONCLUSION

This study provides the first comprehensive characterization of the volatile aroma compounds contributing to the aroma profile of litchi distilled spirit (Heiye). One twenty‐eight different aroma compounds were identified in this study, which belonged to 6 chemical groups, including 39 esters, 16 alcohols, 16 acids, 22 terpenes, 17 aldehydes and ketones, and 18 other compounds. According to the determined concentrations of total aroma components, they were ranked in the following order (highest to lowest concentration): esters; terpenes; acids; alcohols; others, and aldehydes and ketones. The concentrations and kinds of total volatile aroma compounds were higher in distilled spirit than litchi wine. The richest variety of OAVs >1 was also observed in litchi distilled spirit; there were 19 kinds of volatile aroma compounds, but only 16 kinds of them in litchi wine. β‐damascenone, linalool, ethyl butyrate, ethyl isovalerate, ethyl caproate, trans‐rose oxide, and cis‐rose oxide were the essential aroma‐active compounds and played a decisive fruit and flower aroma in litchi distilled spirit. These findings provided comprehensive knowledge on the aroma character of litchi wine and distilled spirit.

## 5 INFORMED CONSENT

Written informed consent was obtained from all study participants.

## CONFLICTS OF INTEREST

The authors declare no competing financial interests.

## AUTHOR CONTRIBUTIONS

**Zhao Lili:** Data curation (equal); Writing‐original draft (lead). **Ruan Shili:** Methodology (equal); Resources (supporting). **Yang XiangKe** **:** Software (lead); Validation (equal). **Chen QiLing** **:** Formal analysis (equal); Resources (lead). **Shi Kan:** Conceptualization (lead); Validation (equal). **Lu Ke:** Data curation (equal); Investigation (lead). **Ling He:** Methodology (equal); Validation (lead). **Yangbo Song:** Writing‐original draft (equal); Writing‐review & editing (lead). **Shuwen Liu:** Project administration (lead); Writing‐review & editing (supporting).

## ETHICAL APPROVAL

This study does not involve any human or animal testing.

## Supporting information

Table S1
