# Supplementary material for: Characterization of volatile aroma compounds in litchi (Heiye) wine and distilled spirit
Source: Food Sci Nutr. 2021 Sep 18;9(11):5914–27. doi: 10.1002/fsn3.2361 (PMC8565214; doi:10.1002/fsn3.2361)
Supplement: Supplementary file 1 — Table S1 [file FSN3-9-5914-s001.docx]

**Supplementary Materials:**

**Supplementary Table 1.** Concentration and OAV Value of Volatile Compounds in Heiye and Guiwei.

| **No.** | **Aroma compound** | **Odor**  **Threshold (μg/L)** | **Concentration (μg/L)** | | **Odor activity value** | | **Odor**  **description** |
| --- | --- | --- | --- | --- | --- | --- | --- |
|  |  |  | Heiye | Guiwei | Heiye | Guiwei |  |
| 1 | Ethyl acetate | 7500 | 49.52 | 78.10 | 0.006 | 0.01 | Sweet, fruity |
| 2 | 1-Pentanol | 80000 | 30.37 | 31.90 | 0.0003 | 0.0004 | Mellow, astringent |
| 3 | 3-Methyl-3-butene-1-ol | 600 | 9.90 | 6.07 | 0.02 | 0.01 | Sweet, fruity |
| 4 | Hexyl alcohol | 8000 | 15.85 | 22.55 | 0.002 | 0.003 | Grassy |
| 5 | 1-Octen-3-ol | 18 | 52.29 | 35.79 | 2.91 | 1.99 | Herbs, mushroom |
| 6 | 2-Ethylhexanol | 900 | 23.11 | 28.28 | 0.03 | 0.03 | undesirable flavours |
| 7 | Octanoic acid | 500 | 71.04 | 84.70 | 0.14 | 0.17 | Cheese, fatty acid |
| 8 | Decanoic acid | 1000 | 50.75 | 54.51 | 0.05 | 0.06 | fatty acid |
| 9 | cis-Rose oxide | 0.2 | 169.34 | 241.66 | 846.70 | 1208.3 | Rose, flower |
| 10 | trans-Rose oxide | 0.2 | 75.32 | 131.67 | 376.63 | 658.35 | Rose, flower |
| 11 | Linalool | 1.5 | 85.18 | 63.25 | 56.78 | 42.17 | Musk, flower, fruit |
| 12 | 4-Terpineol | 110 | 28.84 | nd | 0.26 | nd | Sweet, grassy |
| 13 | p-Menth-1-en-8-ol | 250 | 71.58 | 62.70 | 0.29 | 0.25 | grassy |
| 14 | D-Citronellol | 100 | 263.89 | 488.51 | 2.63 | 4.89 | Citrus, lemon |
| 15 | cis-Geraniol | 300 | 33.12 | 47.36 | 0.11 | 0.16 | Sweet, rose |
| 16 | Geraniol | 5 | 397.44 | 538.45 | 79.49 | 107.69 | Sweet, rose, lemon |
| 17 | cis-citral | 1000 | nd | 58.85 | nd | 0.06 | Fruity, floral |
| 18 | trans-citral | 1000 | nd | 90.75 | nd | 0.09 | Fruity, floral |
| 19 | octanal | 1 | nd | 20.35 | nd | 20.35 | Bitter, lemon |
| 20 | (Z)-2-Nonenal | 13 | nd | 17.60 | nd | 1.35 | Green |
| 21 | trans-2-Hexenal | 17 | 18.56 | 6.63 | 1.09 | 0.39 | Grassy |
| 22 | 6-Methyl-5-hepten-2-one | 50 | 22.45 | 13.69 | 0.45 | 0.274 |  |
